# Quantification of enantiomers and blind identification of *erythro*-sphingosine non-racemates by cold ion spectroscopy†

**DOI:** 10.1039/d4an00911h

**Published:** 2024-08-12

**Authors:** Vladimir Kopysov, Ruslan Yamaletdinov, Oleg V. Boyarkin

**Affiliations:** a SCI-SB-RB Group, ISIC, École Polytechnique Fédérale de Lausanne 1015 Lausanne Switzerland oleg.boiarkin@epfl.ch

## Abstract

Enantiomers of a lipid *erythro*-sphingosine have been quantified with ≈4% accuracy by UV cold ion spectroscopy of their non-covalent complexes with a chiral aromatic molecule. The diastereomeric configuration of such complexes enables the quantification using just a single enantiomeric lipid standard and the identification of non-racemic solutions with no standards at all.

## Abstract 2

UV cold ion spectroscopy quantifies enantiomers using single chiral standard and detects non-racemates having no their standards.

## Graphical abstract

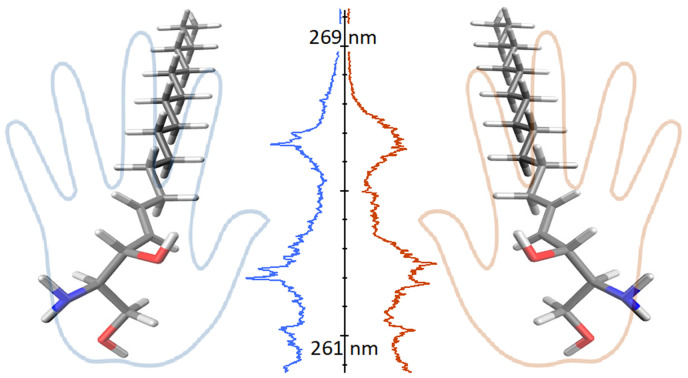

Vast majority of biologically relevant molecules are chiral, that is, non-superposable on their mirror images. Although l/d-enantiomers of chiral molecules have absolutely identical 3D-structure, they interact differently with other chiral molecules. Living organisms demonstrate the dominance of one enantiomeric form over another, a phenomenon called homochirality.^1^ For instance, amino acids predominantly appear in the left-handed form, while monosaccharides – in the right-handed form. Due to the intrinsically chiral environment, enantiomers typically have substantially different biological properties, which makes chiral analysis essential for life sciences and drug design.^2^

Identification of enantiomers, however, remains among the challenging tasks in analytical chemistry. Direct methods for chiral analysis are based on the difference in the interaction of a chiral molecule with left- and right-handed circularly polarized light and include techniques such as electronic and vibrational circular dichroism (CD) and Raman optical activity (ROA) spectroscopy.^3–7^ Alternatively, enantiomers can be differentiated by their interaction with other chiral molecules. For instance, the “gold standard” technique for structural determinations, nuclear magnetic resonance (NMR) spectroscopy, employs chiral derivatizing agents, which chemically react with enantiomers. In addition, chiral solvating or lanthanide shift agents can be used to form non-covalent complexes with enantiomers. Such pairwise produced diastereomers then can be readily discriminated by their NMR spectra.^8,9^ The application of these techniques however is often limited by their relatively low sensitivity. In contrast, mass spectrometry (MS) provides exceptionally high sensitivity and speed of analytical analysis. Although this technique intrinsically cannot distinguish enantiomers, which have exactly the same masses, it can be used as a high-performance detector for a preceding isomer separation technique, such as, for instance, chiral liquid chromatography (LC) or, since recently, ion mobility spectrometry (IMS). Chiral LC is considered as the method of choice for enantiomeric separation, although it requires choosing an appropriate column and a careful optimization of chromatographic conditions.^10^ IMS is often capable of separating structural isomers but was repeatedly reported to fail in resolving enantiomers using 2-butanol as a chiral modifier of the buffer gas.^10,11^ Somewhat better results have been obtained by using IMS for separation of non-covalent diastereomeric complexes formed by enantiomers with chiral additives prior to IMS,^12–14^ although even in this case, the performance of IMS is still inferior to that of LC.

Cold ion spectroscopy (CIS), which is based on the measurement of ultraviolet (UV) or infrared (IR) photofragmentation spectra of cold gas-phase ions, is another recently emerging technique capable of identifying isomers.^15^ Cooling ions to cryogenic temperatures often allows for vibrational resolution in UV and IR spectra, which makes them highly specific to 3D structure of the ions. A library of such spectra recorded for a set of isomers can be used to identify and quantify them in solution mixtures. This approach has been successfully employed for identification of isomeric peptides, oligosaccharides, lipids, and drug molecules.^16–20^ Although enantiomers have absolutely identical optical spectra, they still can be distinguished by CIS, if one makes use of non-covalent binding between a chiral analyte and a suitable chiral “reporter” molecule, which can be added directly into the analyte solution. The 3D structures of such diastereomeric complexes formed with two different enantiomers must be different, potentially, leading to non-identity of their UV/IR spectra. The use of chiral UV-absorbing chromophores for distinguishing enantiomers of biomolecules has been earlier demonstrated for monosaccharides,^17^ which have a large number of hydroxyl groups. Identification and, in particular, quantification of isomeric lipids by means of CIS is more challenging, however, due to their tremendous structural diversity, low number of functional groups, and, often, long unstructured hydrophobic “tails”. Nevertheless, a reliable discrimination of structural isomers of, for instance, steroids, phosphatidylcholines, and prostaglandins has been successfully performed using different pre-selected reporter molecules.^18^ Sphingolipids are another class of lipids, which are one of the main components of mammalian cells membranes and are involved in signal transduction and cell recognition processes. The core part of sphingolipids is sphingosine, a lipid that has two asymmetric carbon atoms and can thus exist as four possible stereoisomers, l/d-*erythro*- and l/d-*threo*-sphingosine. While sphingolipids naturally occur in the d-*erythro* form, a number of studies have shown that their stereoisomers exhibit different physicochemical and biological properties.^21–25^ Recently, IR spectroscopy of non-covalent complexes with the (*S*)-enantiomer of 2-butanol was demonstrated for distinguishing d-*erythro*-sphingosine (d-ES) and its diastereomer, l-*threo*-sphingosine.^20^ Herein, we extend this study and evaluate IR- and UV-based approaches for identification and quantification of the enantiomers of this test lipid. We further demonstrate how UV CIS can be employed for quantification of mixtures of enantiomers in the case, when only one sterically pure isomer is available as a standard. Finally, we propose and test with this lipid an approach for detecting non-racemic mixtures without use of any standards at all.

Our experimental setup has been described elsewhere (see also ESI† for details).^15,26^ Briefly, protonated complexes of the lipids with a UV chromophore or with a solvent molecule are produced from solution by a nano-electrospray ionization (n-ESI) source and selected by a quadrupole mass filter. The complexes are then transferred to a cold octupole ion trap, where they get trapped and cooled down to *T*_vib_ ≈ 10 K upon collisions with He atoms. The cold complexes that contain a UV chromophore or a solvent molecule undergo photofragmentation by a single pulse of a wavelength-tunable UV or IR OPO, respectively. Finally, a quadrupole mass spectrometer detects alternately the precursor complexes and protonated ES at each wavelength of the OPO.

Fig. 1 shows the recorded IR spectra of the complexes of l-ES and d-ES lipids with the (*S*)-enantiomer of 2-butanol in the frequency region of OH/NH-stretch vibrations, for which commercial laser sources are readily available. Surprisingly, and in contrast to the spectra of d-ES and its diastereomer l-*threo*-sphingosine,^20^ the spectra of the complexes with l-ES and d-ES look very similar. Above 3600 cm^−1^ the two spectra exhibit three broad bands, which are resolved and readily assigned to the OH stretches of the complexes. The three bands that appear in the region of NH stretches (3200–3300 cm^−1^) are even broader but still remain vibrationally resolved. Despite the resolution, the IR spectra of the complexes of l-ES and d-ES with 2-butanol in Fig. 1 demonstrate only a slight difference in the relative intensities and shapes of the bands, while the frequencies of the band maxima are the same. This makes an accurate quantification of the ES enantiomers impractical. Quantum chemical calculations of the 3D structure and IR spectra of the complexes, which are still difficult for such a flexible system, may reveal the origin of this spectral similarity and, perhaps, suggest the spectral regions, where the difference might be substantial.

**Fig. 1:**
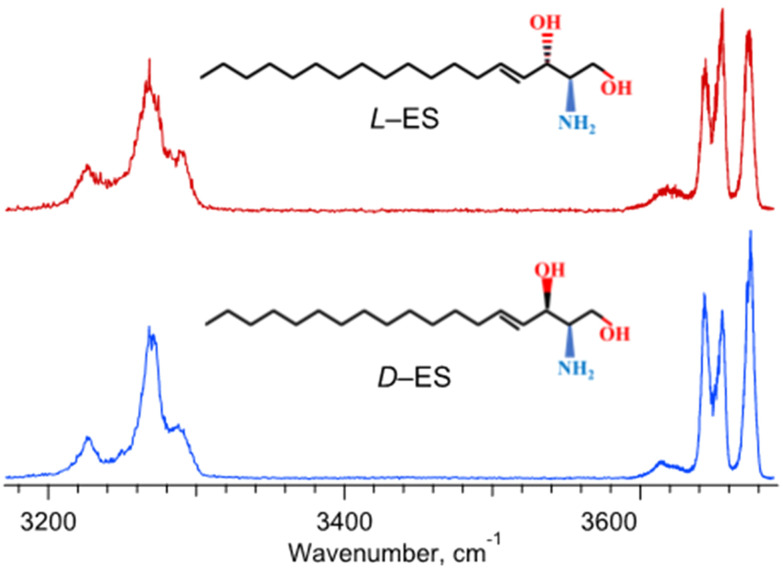
IR photofragmentation spectra of the protonated non-covalent complexes of l-ES and d-ES enantiomers (red and blue traces, respectively) with (*S*)-2-butanol.

An alternative approach for identification of enantiomers of a UV non-absorbing analyte (like ES lipid here) by CIS implies UV photofragmentation of non-covalent diastereomeric complexes formed in solution (or in ESI droplets) between the analyte molecule and a suitable chiral UV chromophore. Readily available chromophores such as tyrosine (Tyr) and phenylalanine (Phe) appeared to work equally well for identification of, for instance, isomeric glycans and enantiomeric monosaccharides, respectively.^17^ In contrast, the structural diversity of lipids requires a pre-selection of appropriate UV chromophores, which are specific for lipids of different types and sizes. Our previous study suggested a tentative guide for this selection.^18^ For structural isomers that involve long nonpolar chains with no functional groups, a good sensor should have no polar groups on its aromatic ring and own some flexibility of the ring relative to the point of attachment. This makes, for instance, aromatic dipeptide Phe-Phe (Phe_2_) particularly sensitive to structural differences of isomeric phospholipids.^18^

The UV spectra of the complexes of l-ES and d-ES with all-L dipeptide (l-Phe)_2_ (Fig. 2a), indeed, exhibit distinct shapes in the region of the absorption onset, as well as a number of spectral features specific to the enantiomers (*e.g.*, at 262.4 and 262.98 nm for l-ES and at 262.57 and 262.85 nm for d-ES). To evaluate the accuracy of the library-based quantification of the ES enantiomers in their solution mixtures, we prepared a set of 7 mixtures with the relative concentrations of the enantiomers ranging from 0 to 100%, added (l-Phe)_2_ into each solution, and recorded UV spectra of the electrosprayed complexes. Numerical decomposition of the spectra of the mixtures over the library spectra of the pure enantiomers reproduced their relative concentrations in the mixtures with the root-mean-square (RMS) error of 3.6% (Fig. S1a†).

**Fig. 2:**
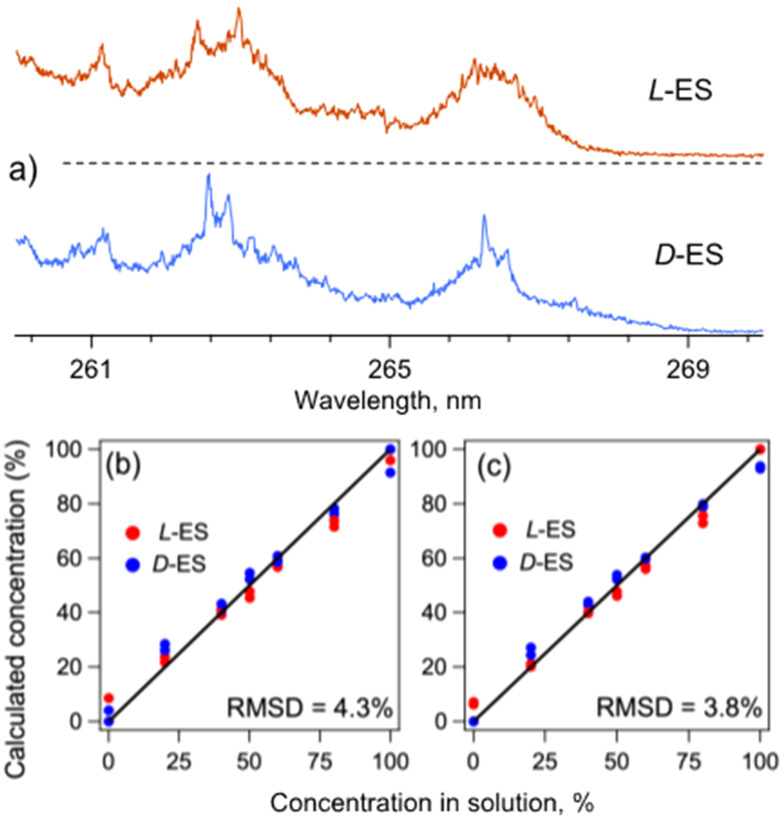
(a) UV photofragmentation spectra of (l-ES)–(l-Phe)_2_ and (d-ES)–(l-Phe)_2_ protonated non-covalent complexes (red and blue traces, respectively), measured by detecting all abundant photofragments (assigned as protonated Phe_2_, ES and ES with water loss; Fig. S5†). (b and c) Calculated relative concentrations of l-ES and d-ES (red and blue dots, respectively) as a function of their relative concentrations in 14 solution mixtures. The concentrations were calculated using the UV spectra of the complexes of either (b) (l-Phe)_2_ with both enantiomers of ES or (c) l-ES with both enantiomers of Phe_2_. The solid lines show the expected 1 : 1 dependency.

Instead of (l-Phe)_2_, one can equally use its all-D enantiomer (d-Phe)_2_, which is also readily commercially available, and the same library spectra measured with (l-Phe)_2_ for quantification of the analyte mixtures. Indeed, because of the symmetry, the (l-Phe)_2_–(l-ES) and (l-Phe)_2_–(d-ES) complexes must be enantiomers of the (d-Phe)_2_–(d-ES) and (d-Phe)_2_–(l-ES) complexes, respectively. That is, simultaneous inversion of the chirality (d ↔ l) of an analyte and a reporter molecule, fundamentally, does not change the geometry of their complex and, therefore, its spectrum. To illustrate this symmetry property, we recorded UV spectra of the (d-Phe)_2_–(l-ES) and (d-Phe)_2_–(d-ES) complexes. These spectra appear, indeed, to be identical to those of the (l-Phe)_2_–(d-ES) and (l-Phe)_2_–(l-ES) complexes, respectively (Fig. S2†). Regarding this, we repeated the measurements with the same as above 7 test mixtures, but this time with (d-Phe)_2_ reporter molecule. The numerical analysis yielded the RMS error of 4.8% for the calculated relative concentrations of the enantiomers of ES, such that the overall error for the set of the 14 mixtures becomes 4.3% (Fig. 2b).

The described above unique d ↔ l symmetry property of non-covalent complexes formed by chiral molecules allows for library-based quantification of enantiomers even when only one of them is available as a sterically pure standard (*e.g.*, a product of an enantioselective enzymatic reaction). In such a case, the two library spectra can be obtained by performing measurements for the complexes of this single standard with two enantiomers of a chiral reporter chromophore. Because of the d ↔ l property, the two measured spectra will represent what could be measured for both enantiomers of the analyte but with (any) one of the two enantiomers of the reporter.

As an example, we calculated relative concentrations of l/d-ES in the same set of 14 mixtures using the UV spectra of the (l-Phe)_2_–(l-ES) and (d-Phe)_2_–(l-ES) complexes as the library. The resulting RMS error of 3.8% (Fig. 2c) is slightly smaller than in the case of the use of the library recorded only with (l-Phe)_2_ but with both enantiomers of ES. The difference, likely, reflects a non-ideal reproducibility of the measurements (Fig. S3†). Averaging the library spectra for each pair of the enantiomeric complexes reduces the RMS error of ES quantification to 2.7% (Fig. S1b†). This result suggests that the error can be, potentially, further reduced by improving the signal-to-noise ratio of the data.

The demonstrated above library-based approach for quantification of enantiomers still requires at least one enantiomerically pure standard of an analyte molecule. Such standards, however, can be either unavailable or simply unknown, for instance, in the case of a chemically unidentified yet metabolite. What information about the isomeric composition CIS can provide in such a “blind” case? The number, the UV and photofragment mass spectra of isomers of any type (*e.g.*, functional, positional, *etc*.) that are present in a sample mixture, potentially, can be determined by the method of 2D UV-MS fingerprinting of cold ions.^16^ This, however, requires not only the UV spectra but also the fragment MS spectra of all the isomers to be substantially different, which is, apparently, not the case for enantiomers. Nevertheless, UV sensing of non-covalent complexes of a chiral analyte with a suitable chiral chromophore can identify non-racemic analyte mixtures. Indeed, one can compare the UV spectra measured for an unknown sample using l- and d-enantiomers of a chiral messenger. If the two spectra are different, the sample contains a non-racemic mixture of enantiomers (or a pure enantiomer). If, however, the measured spectra are identical, either the mixture is racemic or the spectra of the complexes with the pure enantiomeric standards are almost indistinguishable. This conclusion remains valid even if there are enantiomers of more than one structural isomer: the method determines the total non-racemic balance of the sample for all the isomers that bind to the messenger molecule.

As an example, Fig. 3 shows the pairs of UV spectra measured for 7 test mixtures of l/d-ES probed with (l-Phe)_2_ and (d-Phe)_2_ chromophores; the pairs correspond to (l-ES)/(d-ES) mixtures with different relative concentrations. As expected, the UV spectra of the complexes for the solutions with a pure ES enantiomer (top and bottom pairs of traces) are visually different and therefore can be confidently assigned to non-racemic samples. The difference becomes less evident, however, upon approaching to the racemic mixture. In practice, even the spectra that are repetitively remeasured for the same sample never appear ideally identical due to unavoidable experimental errors and noise (Fig. S3†). In such cases, the decision on whether the spectral difference exceeds the experimental errors or not can be made on the basis of statistical hypothesis testing. For instance, one can assess the identity of two spectra at each wavelength using the Welch's *t*-test.^27^ To perform the test, we recorded each of the 14 spectra in Fig. 3 four times and, at each wavelength, calculated a, so-called, *p*-value, which, roughly, can be considered as a probability for two signals to be the same (see ESI† for details). For instance, at the wavelengths *λ* that are shown in Fig. 3 by grey lines, *p*-value(*λ*) < 0.005 (*i.e.*, 0.5%, one of the standard values used for statistical tests). That is, within the frame of our test and for the achieved experimental accuracy, the fragmentation yields for these mixtures are confidently unequal at these wavelengths.

**Fig. 3:**
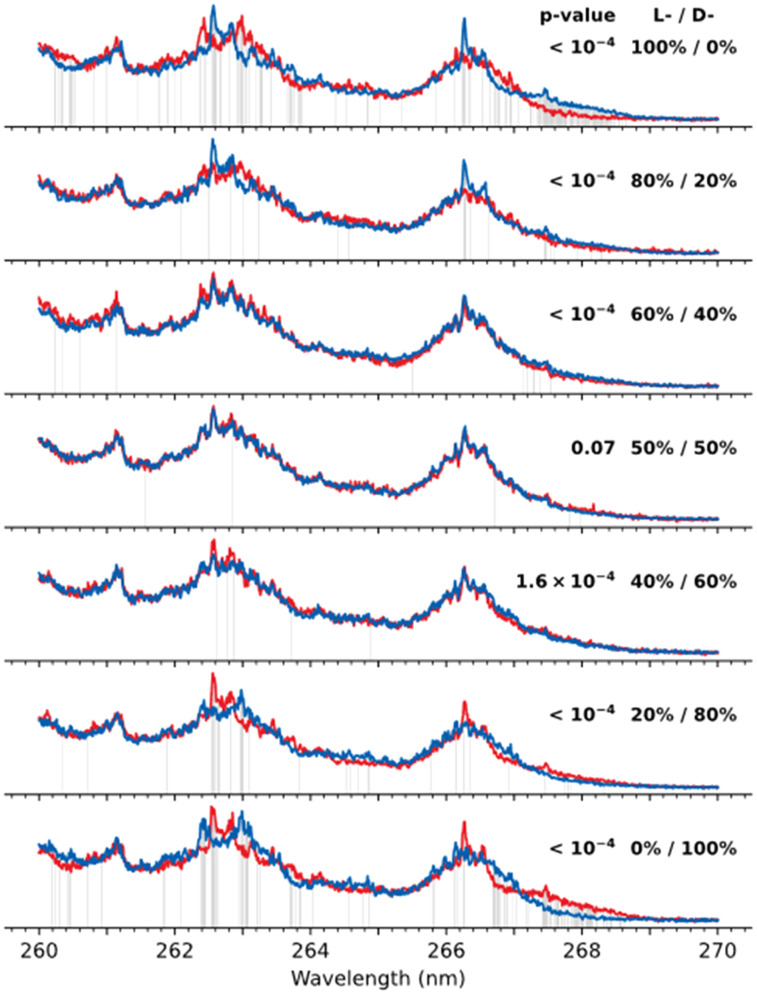
UV spectra (averaged over multiple measurements) of mixtures of l-ES and d-ES enantiomers probed by adding to the mixtures either (l-Phe)_2_ (red trace) or (d-Phe)_2_ (blue trace) chromophore. Each pair of the spectra is labelled by the relative concentrations of l/d-ES lipids. The grey lines highlight the wavelengths at which the spectra were assessed as being different (*p*-value < 0.005) according to the Welch's *t*-test; the shown *p*-values are the results of the Fisher's combined probability test.

A tunable laser source, like in our experiments, makes the measurements more efficient and reliable, since it increases the chance to come across the wavelengths at which the fragmentation yields of the complexes with two enantiomers of a chromophore are substantially different. Moreover, one can compare the spectra as a whole using, for instance, the Fisher's combined probability test.^28^ It allows aggregating the *p*-values calculated at each wavelength into a single metric that assesses the identity of the two spectra. The resulting *p*-values of the Fisher's test, shown in Fig. 3, indicate that the pairs of spectra for all the mixtures, except for the racemic one, should be considered as non-identical at the significance level of 0.005, which means that these mixtures are non-racemic. To get a sense, how quickly the *p*-value may change upon enantiomeric excess in a sample, we have done a numerical modelling with our data for the enantiomers of ES (see ESI† for details). It shows that the *p*-value exhibits a threshold-like drop above ∼15% of enantiomeric excess and quickly drops below 10^−4^ already for the 18% excess (Fig. S4†). It is worth noting that, because the individual spectra of the complexes with two enantiomeric analytes are unknown, the described above tests fundamentally cannot quantify their mixtures, and a low *p*-value should be considered only as a qualitative indicator of a non-racemic sample.

In the case of the racemic mixture, the test qualifies the spectra as indistinguishable. However, this could happen not only because a mixture is racemic, but also if a chromophore is not selective enough to discriminate the enantiomers of an analyte. Therefore, the test gives a definite answer only if the resulting *p*-value appears to be lower than a chosen significance level. Fundamentally, the structures and therefore the spectra of diastereomeric complexes cannot be exactly the same. This implies that, in principle, any non-racemate can be detected by the proposed method – a subject of experimental accuracy. One may draw a certain similarity of this spectroscopic approach with LC or IMS separations. The two enantiomers or their complexes with a chiral reporter can be time-separated by LC and IMS, respectively, only if the resolution is sufficiently high to detect two peaks, otherwise the test remains uncertain. Increasing the resolution in these two methods implies, for instance, longer/repetitive measurements and selection of the LC column or of the reporter molecule. Similarly, the spectroscopic method requires repetitive measurements and selection of a reporter molecule too.

In conclusion, we tested IR/UV cold ion spectroscopy of non-covalent complexes for library-based quantification of the enantiomers of *erythro*-sphingosine lipid as an example. High similarity between the IR spectra of l- and d-ES enantiomeric ions tagged by chiral (*S*)-2-butanol solvent hinders their use for distinguishing the enantiomers. On the contrary, UV CIS of the complexes of l/d-ES with (l-Phe)_2_ chiral chromophore allows for quantification of the enantiomeric composition of the ES solution samples, on average, with 2.7% accuracy. Moreover, the fundamental d ↔ l symmetry property of non-covalent complexes of analyte–chromophore chiral molecules enables recording the library using only one enantiomer of the analyte but both enantiomers of the chromophore instead.

Finally, we have demonstrated a new approach that allows for detection of non-racemic solution samples even when none of the two enantiomeric standards of the analyte is available. This requires multiple measurements of the spectra for the complexes of the analyte with l- and d-chromophore. If the difference between the two averaged spectra is substantially higher than the experimental error, one can be confident that the sample is non-racemic. Such a “blind” test, potentially, can be done even at a single wavelength of a non-tunable UV laser (*e.g.*, at 266 nm, the fourth harmonic of Nd:YAG laser) but, perhaps, with many repetitive measurements and/or with a set of different chromophores.

Although demonstrated for ES, the developed herein approach for enantiomeric identifications, potentially, can be applied to other lipids and, more generally, to different types of chiral biomolecules and drugs. The “blind” detection of non-racemic samples does not require any prior knowledge of the sample structure/composition and, potentially, can be used to determine the presence of an overall enantiomeric excess for a mixture of a few structurally different isomers.

## Data availability

The datasets supporting this article have been uploaded as part of the ESI.†

## Conflicts of interest

There are no conflicts to declare.

## Supplementary Material

AN-149-D4AN00911H-s001
