# Supplementary material for: Quantification of enantiomers and blind identification of erythro-sphingosine non-racemates by cold ion spectroscopy
Source: Analyst. 2024 Aug 12;149(18):4600–4. doi: 10.1039/d4an00911h (PMC11323735; doi:10.1039/d4an00911h)
Supplement: AN-149-D4AN00911H-s001 [file AN-149-D4AN00911H-s001.pdf]

Supplementary Information (SI) for Analyst.
This journal is © The Royal Society of Chemistry 2024

Quantification of Enantiomers and Blind Identification of Erythro-Sphingosine Non-Racemates by Cold Ion Spectroscopy.

Vladimir Kopysov,[a] Ruslan Yamaletdinov [a] and Oleg V. Boyarkin *[a]

SCI-SB-RB, ISIC, École Polytechnique Fédérale de Lausanne, CH-1015 Lausanne, Switzerland

* Correspondence to: oleg.boiarkin@epfl.ch

Materials and Methods S2

Chemicals S2

Experimental method S2

Statistical hypothesis testing S2

Data analysis S3

Fig. S1 Calculated relative concentrations of L/D-ES S4

Fig. S2 UV spectra of L/D-ES with (L/D-Phe)$_2$ S4

Fig. S3 Repetitive measurements of the UV spectra of the racemic mixture of ES with (L/D-Phe)$_2$ S5

Fig. S4 p-values as a function of the enantiomeric excess S6

Fig. S5 Photofragmentation mass spectrum of L-ES with (L-Phe)$_2$ S6

Materials and Methods

Chemicals

L- and D-*erythro*-sphingosines (≥98% purity) were purchased from Cayman Chemical; L-Phe-L-Phe and D-Phe-D-Phe (≥98% purity) were purchased from Bachem; water and acetic acid (Optima™, LC-MS grade) were purchased from Fisher Scientific; isopropyl alcohol (CHROMASOLV™, LC-MS grade) was purchased from Honeywell; and (*S*)-2-butanol (≥98.5% purity) was purchased from Thermo Scientific.

Individual stock solution of diphenylalanine (Phe_2) and *erythro*-sphingosines (ES) were prepared in water and isopropyl alcohol, respectively. All the solutions for UV spectroscopy measurements had the following composition: 50 μM of Phe_2 (either $(L\text{-}Phe)_2$ or $(D\text{-}Phe)_2$) and 50 μM of ES (total concentration of both enantiomers, while the enantiomeric excess was varied) in a mixture of water and isopropyl alcohol (50:50) with 1% of acetic acid. For IR spectroscopy measurements, an aliquot of the stock solution of one of the ES enantiomers was diluted to a concentration of 100 μM in (*S*)-2-butanol with 1% of acetic acid.

Experimental method

Protonated complexes of ES either with an (*S*)-2-butanol molecule or with a UV chromophore are produced from solution by a nano-electrospray ionization (nano-ESI) source and transferred through a metal capillary and three consecutive coaxial molecular skimmers to a linear octupole trap (pre-trap). After accumulation and thermalization in the pre-trap, the complexes are released, mass-selected by a quadrupole mass filter, turned by 90° using an electrostatic bender, focused by a stack of electrostatic lenses, and moved through an RF octupole guide into a cold octupole trap kept at 6 K. The trap is driven by two 1 MHz sinus waveforms with peak-to-peak amplitudes of 50-100 V. The complexes get trapped and cooled down to $T_{vib} \approx 10$ K upon collisions with a He buffer gas, which is pulsed into the trap shortly before the arrival of the ions. The cold complexes are then irradiated by a pulse of UV or IR light. The UV light (≈2 mJ/pulse, ≈5 ns duration, ≈6 cm^{-1} spectral linewidth) is produced by a UV optical parametric oscillator (OPO; NT 342C, EKSPLA), and the IR light (≈4 mJ/pulse, ≈1 cm^{-1} spectral linewidth) is produced by a LaserVision IR OPO pumped by a Nd:YAG laser (InnoLas SpitLight 600). The resulting parent and fragment ions are released from the trap, turned by 90° using the second electrostatic bender, and detected by the second quadrupole mass filter equipped with a channeltron detector. While the laser pulses are generated at a 10 Hz repetition rate, the mass spectrometer operates in a 20 Hz cycle. This allows recording the signal of the parent ions in the laser-free cycle, which is used to normalize the signal of the fragment ions. The photofragmentation yield (the ratio of the number of the fragment and parent ions) is then normalized by the laser pulse energy, measured by a broadband pyroelectric detector. To improve the signal-to-noise ratio, the parent and fragment signal as well as the laser pulse energy are averaged over 10 measurements at each wavelength prior to normalization.

Statistical hypothesis testing

First, consider the data recorded at a certain wavelength. For a mixture with L- and D-chromophores, the photofragmentation yields can be represented by two sets of numbers $\{x_1, x_2, \dots, x_n\}$ and $\{y_1, y_2, \dots, y_m\}$, where n and m are the number of repetitive measurements. One can characterize these two samples by their sample means, $\bar{x}$ and $\bar{y}$, and by their corrected sample standard deviations, s_x and s_y. To test the null hypothesis that the populations, from which the samples were drawn, have equal means, the Welch's *t*-test defines the following *t* statistics:

$$t = \frac{\bar{x} - \bar{y}}{\sqrt{s_x^2/n + s_y^2/m}}$$

Knowing the statistical distribution of the test statistics and having its value calculated for the observed data, one can evaluate the so-called *p*-value. This value is the probability under the null hypothesis of obtaining the test statistics at least as extreme as the actually observed value. In other words, when *p*-value is small, the null hypothesis (i.e., the spectra are identical at the selected wavelength) is unlikely to be true. The lower the *p*-value, the stronger the evidence against the null hypothesis. Finally, to make a binary decision (i.e., true or false) about the validity of the

null hypothesis, one needs to choose a significance level (α). If the *p*-value is less than the significance level, the null hypothesis is rejected. With respect to the spectral data, this would mean that, at the considered wavelength, the spectra statistically are not identical and therefore cannot correspond to a racemic mixture.

In order to compare the spectra of the mixture with L- and D-chromophores as a whole, one could perform a true multivariate test, such as the Hotelling's *T*-squared test, which is a generalization of the Student's *t*-test for multivariate data. This, however, would require recording an impractically large number of spectra, at least, the same as the number of wavelengths. Instead, we employed the Fisher's combined probability test, which allows aggregating the *p*-values of several independent tests into a single statistic:

$$X_{2k}^2 = -2\sum_{i=1}^{k} \ln p_i$$

where k is the number of tests, that is, the number of wavelengths in the spectra. Thus, by calculating the *p*-values at each wavelength using the Welch's test and aggregating them into X_{2k}^2, one can evaluate the *p*-value regarding the null hypothesis that the spectra of the mixture with L- and D-chromophores are statistically the same. If the resulting *p*-value is less than a preliminary chosen significance level, one can conclude that the spectra are different and cannot correspond to a racemic mixture.

Data analysis

Processing of the raw experimental data and their subsequent analysis were performed using custom scripts written in Python. For the library-based quantification of the ES enantiomers in their mixtures, we employed non-negative least squares optimization implemented in the SciPy package (the *nnls* function from the *optimize* module). For the Welch's *t*-test, *p*-values were calculated using the *ttest_ind* function (with argument *equal_var* set to *False*); for the Fisher's combined probability test, *p*-values were calculated using the cumulative distribution function for a chi-squared distribution generated by the *chi2.cdf* method. Both *ttest_ind* and *chi2.cdf* are implemented in the *stats* module of SciPy package.

To evaluate how the *p*-value of the Fisher's combined probability test depends on the enantiomeric excess of a mixture of L/D-ES, we performed the following numerical simulation. First, we employed the experimentally measured UV spectra of mixtures of pure L-ES with pure $(L\text{-}Phe)_2$ and pure D-ES with pure $(D\text{-}Phe)_2$ to calculate the mean value and the standard deviation at each wavelength. We thus obtained an estimate of the UV spectrum ($m_1(\lambda)$) of this non-covalent complex (the all-L and all-D complexes are enantiomers) and the corresponding experimental noise ($\sigma_1(\lambda)$). Similarly, we evaluated the UV spectrum ($m_2(\lambda)$) and the noise ($\sigma_2(\lambda)$) for the diastereomeric complex using the UV spectra of mixtures of L-ES with $(D\text{-}Phe)_2$ and D-ES with $(L\text{-}Phe)_2$. For a mixture of L/D-ES with a certain enantiomeric excess (ee), we model its UV spectra as:

$$s_1(\lambda) = \frac{1+ee}{2} \cdot \mathcal{N}\left(m_1(\lambda), \sigma_1^2(\lambda)\right) + \frac{1-ee}{2} \cdot \mathcal{N}\left(m_2(\lambda), \sigma_2^2(\lambda)\right)$$

$$s_2(\lambda) = \frac{1-ee}{2} \cdot \mathcal{N}\left(m_1(\lambda), \sigma_1^2(\lambda)\right) + \frac{1+ee}{2} \cdot \mathcal{N}\left(m_2(\lambda), \sigma_2^2(\lambda)\right)$$

If the mole fraction of L-ES is larger than that of D-ES, $s_1(\lambda)$ and $s_2(\lambda)$ correspond to the spectra measured using $(L\text{-}Phe)_2$ and $(D\text{-}Phe)_2$, respectively. Otherwise, $s_1(\lambda)$ and $s_2(\lambda)$ correspond to the spectra measured using $(D\text{-}Phe)_2$ and $(L\text{-}Phe)_2$, respectively. For the Fisher's test, however, the exact assignment of the spectra does not matter, and the result is determined only by the value of enantiomeric excess. To calculate the *p*-value, we performed the test using a set of n spectra $\left\{s_1^{(i)}: i = 1..n\right\}$ and a set of m spectra $\left\{s_2^{(i)}: i = 1..m\right\}$, each of which was independently generated as described above. For each value of enantiomeric excess, we repeated the test 1000 times, each time randomly choosing n and m from a set of integers between 3 and 10.

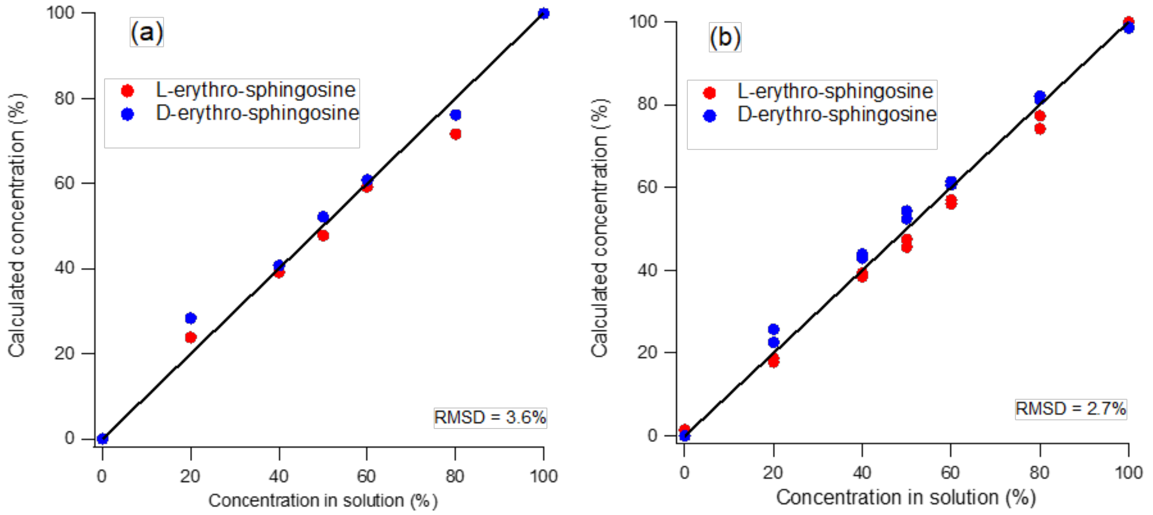

Fig. S1 (a) Calculated relative concentrations of L-ES and D-ES (red and blue dots, respectively) as a function of their relative concentrations in 7 solution mixtures. The concentrations were calculated using the library of the UV spectra of the complexes of (L-Phe)$_2$ with both enantiomers of ES. (b) Calculated relative concentrations of L-ES and D-ES (red and blue dots, respectively) as a function of their relative concentrations in 14 solution mixtures. For the library, we averaged the UV spectra of the following two pairs of enantiomeric complexes: (L-Phe)$_2$–(L-ES)/ (D-Phe)$_2$–(D-ES) and (L-Phe)$_2$–(D-ES)/ (D-Phe)$_2$–(L-ES).

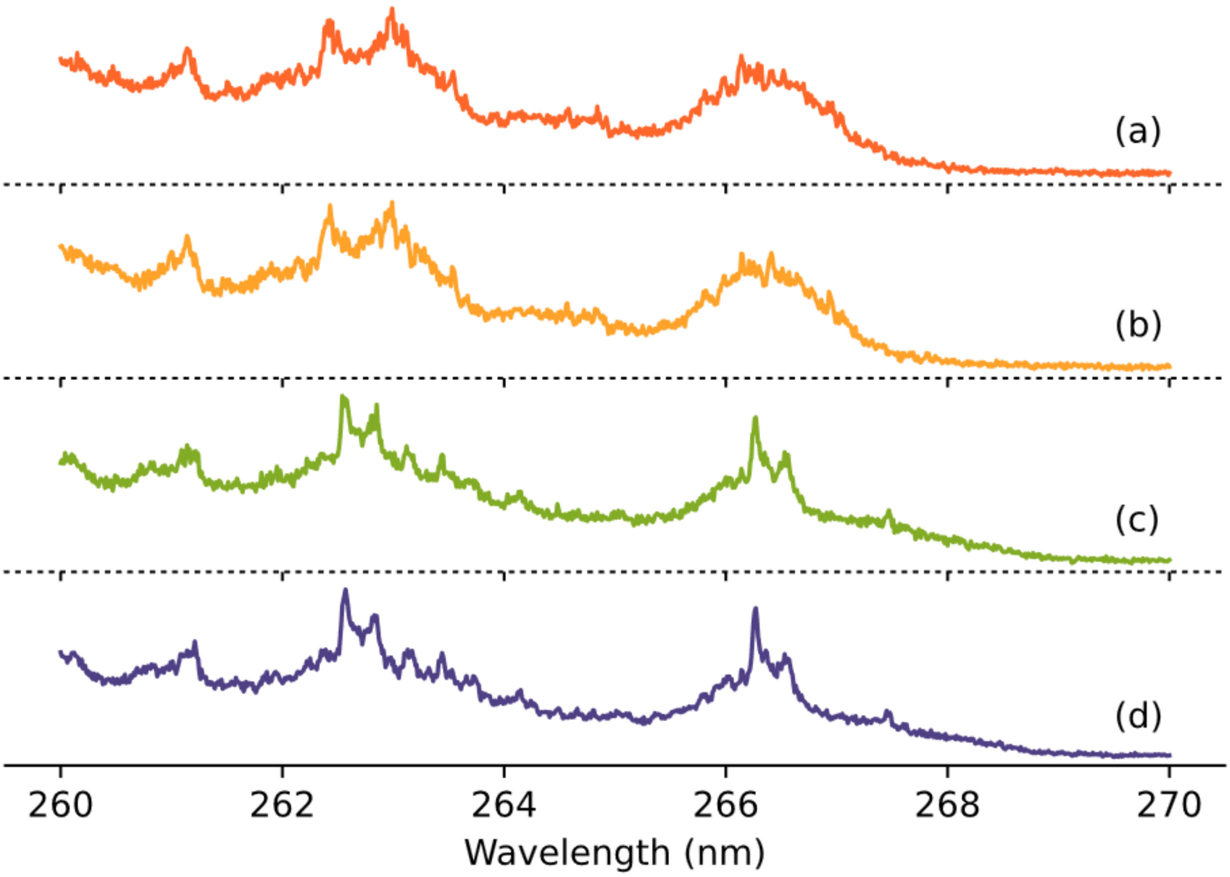

Fig. S2 Photofragmentation UV spectra of noncovalent complexes (a) D-ES–(D-Phe)$_2$, (b) L-ES–(L-Phe)$_2$, (c) D-ES–(L-Phe)$_2$ and (d) L-ES–(D-Phe)$_2$. Note the pairwise similarity of the spectra (a)-(b) and (c)-(d).

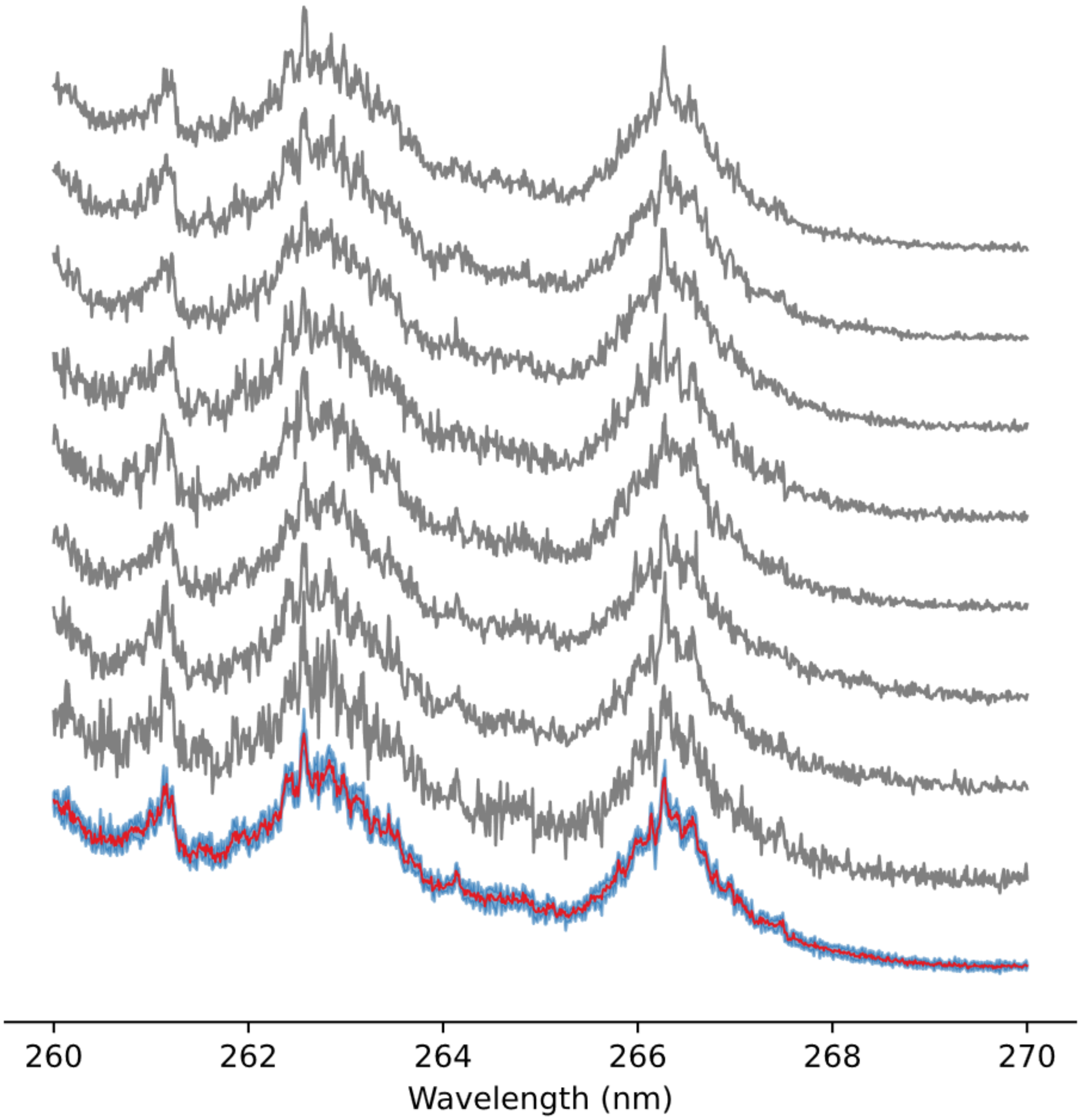

Fig. S3 Photofragmentation UV spectra of the racemic mixture of L-/D-ES (grey traces) measured 4 times using (L-Phe)$_2$ as a reporter molecule and 4 times using (D-Phe)$_2$. The resulting average spectrum is show in red, and the blue area around it indicates the level of experimental noise (± standard deviation).

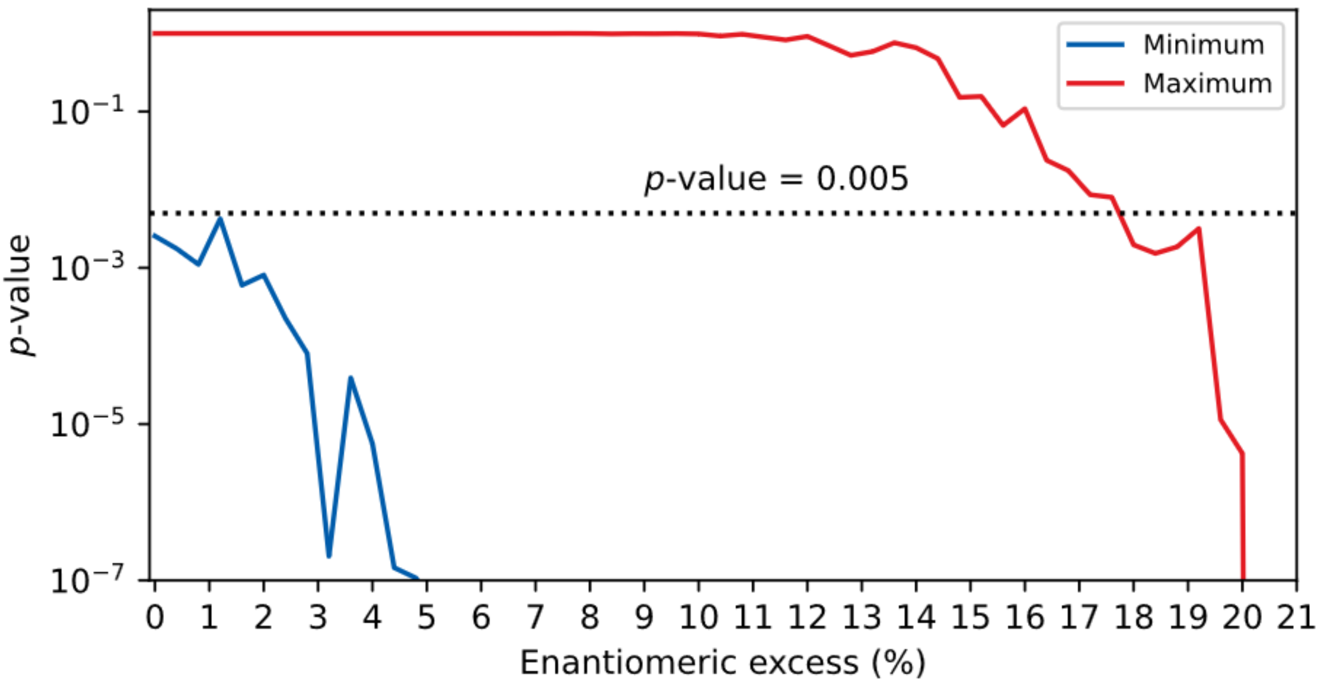

Fig. S4 Evaluated minimum and maximum *p*-values for the Fisher's combined probability test as a function of enantiomeric excess of a mixture of L-/D-ES.

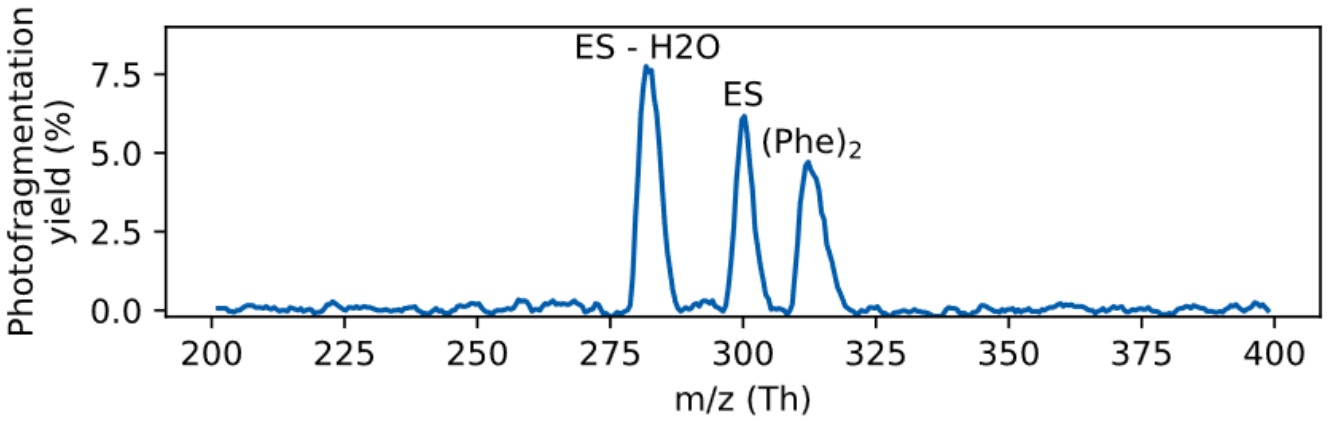

Fig. S5 Photofragmentation mass spectrum of the singly protonated cold non-covalent complexes of L-ES with (L-Phe)$_2$ dipeptide. Photofragmentation was performed at 266.26 nm.
